# Preliminary indoor evidences of microplastic effects on freshwater benthic macroinvertebrates

**DOI:** 10.1038/s41598-020-80606-5

**Published:** 2021-01-12

**Authors:** Luca Gallitelli, Alessandra Cera, Giulia Cesarini, Loris Pietrelli, Massimiliano Scalici

**Affiliations:** 1 Department of Sciences, University of Roma Tre, Viale G. Marconi 446, 00146 Rome, Italy; 2 Department of Chemistry, Sapienza University of Rome, P.le A. Moro, 5, 00185 Rome, Italy

**Keywords:** Ecology, Environmental sciences

## Abstract

Plastics are to date considered one of the main detrimental drivers for the health of aquatic ecosystems, both in marine and inland waters. Regarding the latter habitat, it seems surprising how the plastic effects on benthic invertebrates are neglected since macroinvertebrates have a long tradition in the water quality assessment activities. In this context, we propose timely indoor observations on the exposure of caddisfly *Odontocerum albicorne* and mayfly *Ephemera danica* to various microplastic polymers (ABS, PET, PP, PS, PVDF). Three different experimental designs were performed on caddisflies and mayflies by exposing their larvae to natural and microplastic substrates. Our findings highlighted how microplastics affected both caddisflies in rebuilding its own case (after having removed the natural one) and mayflies burrowing. Particularly, all caddisflies rebuilt cases using the microplastic polymers provided instead of natural items only. Moreover, we provide the first evidence that mayflies burrow mainly in microplastic substrates rather than in natural ones. Our research highlights that macroinvertebrate larvae would use naturally occurring microplastics and this could be of particular concern in freshwaters with high contamination by plastics. Indeed, larvae appear to not necessarily perceive microplastics as a direct stressor. Further studies ought to be conducted to understand the chronic perturbation on larvae fitness and for example, on drift behaviour. Also, further investigations are needed to understand the potentialities of using plastics by benthic macroinvertebrates.

## Introduction

Plastics are synthetic organic polymers whose production is steadily increasing throughout recent years^1^, becoming an issue of growing concern in the Anthropocene, with fly ash, radionuclides, metals, pesticides and greenhouse gases^2^. In particular, microplastics (MP) range from 1 µm to 5 mm and can be subdivided into large MP (lMP, 1–5 mm) and small MP (sMP, 1 µm–1 mm)^3,4^. Moreover, MP can be classified into primary and secondary depending on their origin. Primary MP are industrially manufactured, such as microbeads used in cosmetic products (scrubs, toothpaste, etc.), while secondary MP originate from the fragmentation of larger plastic items, for example due to the environmental exposure (*i.e.* physical, chemical or biological degradation) of litter in water^5^. MP are a highly variable class of contaminants, which are characterised by different shapes and colours^6^. The most common shapes are fragments (three-dimensional shape), films (thin and flat foil), fibres (linear), beads and foams^7^.

MP are widespread worldwide in all systems: from aquatic to terrestrial, including atmospheric one^8^. In aquatic ecosystems, most of the studies have focused on marine environment rather than freshwaters^9–11^. Despite this, it has been highlighted that freshwaters (*i.e.* rivers) are among the main pathways of plastic release to the oceans^12,13^. MP enter freshwater systems through diffuse (wind deposition and run-off) or point (wastewater treatment plants and improper disposal of litter) sources^8^.

The interactions between organisms and MP are deeply investigated in marine habitats rather than in freshwaters^14^. The biological and ecological effects of MP in aquatic environments are diverse: the ingestion and consequent internal physical^15^ and chemical damage^16,17^; trophic transfer and biomagnification^18^; absorption to MP surfaces of other contaminants such as heavy metals^19^, antibiotics^20,21^, polycyclic aromatic hydrocarbons^22,23^ and polychlorinated bisphenols^24^; transport of pathogens and alien species that colonize MP surface^25,26^.

Similar effects can be expected for inland water dwelling taxa^10^. To date, field studies on the relationship between biota and MP in freshwaters have focused mainly on ingestion by fish^27–29^, birds^30–33^, amphibians^34^, crustaceans^35–37^, mollusks^34,38–41^, worms^42^ and hexapods^37,43,44^. Effects on organisms to MP exposure in laboratory were also investigated, such as in *Danio rerio* (Hamilton, 1882)^45–47^ and *Daphnia magna* (Straus, 1820)^48–50^.

Macroinvertebrates are a heterogeneous and not phylogenetic group comprising different taxa, including aquatic insects^51^. Although macroinvertebrates are important bioindicators for water quality (European Water Framework Directive 2000/60/EC) and they have a relevant ecological role, being at the base of trophic webs^52^, it is surprising how few studies are carried out on MP effects on freshwater macroinvertebrates in nature and indoor.

In nature, MP are found in about 50% of the mayflies (Heptageniidae and Baetidae) and caddisflies (Hydropsychidae) analysed in rivers across South Wales, with a maximum concentration of 0.14 MP mg tissue^−1^^44^. Heptageniidae and Hydropsychidae are employed to assess the MP contamination due to point source in River Kinnickinnic in western Wisconsin^37^. In addition, two studies have highlighted that caddisfly specimens can incorporate MP in their case. However, the first study was an occasional observation of one caddisfly case not identified^53^, while the second thoroughly described MP occurrence in natural cases of *Lepidostoma basale* (Kolenati, 1848) specimens^54^.

In indoor experiments, six species are investigated in a recent study: the amphipods *Gammarus pulex* (Linnaeus, 1758) and *Hyalella azteca* (Saussure, 1858), the isopod *Asellus aquaticus* (Linnaeus, 1758), the bivalve *Sphaerium corneum* (Linnaeus, 1758), the worms *Lumbriculus variegatus* (Müller, 1774) and *Tubifex* spp. (Lamark, 1816). They are exposed at realistic concentrations of polystyrene (PS) mixed with sediment. MP exposure caused no effects on mortality of six species. Moreover, no significant effects in growth were observed for *H. azteca, A. aquaticus, S. corneum, L. variegatus,* and *Tubifex spp.* Instead, *G. pulex* showed reduction in growth and MP uptake proportional with MP concentrations in sediment^55^. In addition, two studies were conducted on MP in caddisflies: the first one assessed lethal effects of MP on *Sericostoma pyrenaicum* (Pictet, 1865) at high concentration, *i.e.* 10^3^ particles ml^−1^ ^56^ and the second one highlighted how polyvinyl chloride (PVC) and polyethylene terephthalate (PET) MP reduced case stability in *L. basale*^57^.

In this study we wanted to preliminary analyse how MP affect the behaviour of riverine benthic invertebrates by (i) investigating the ability of caddisfly larvae to use different MP polymers for making the cases, and (ii) assessing whether the mayfly larvae use a MP substrate for burrowing.

## Materials and methods

### Model organisms and sampling activities

We investigated two macroinvertebrate species of two orders: the caddisfly *Odontocerum albicorne* (Scopoli, 1763) (Trichoptera) and the mayfly *Ephemera danica* (Müller, 1764) (Ephemeroptera). The caddisfly larvae builds a diagnostic case with gravel and sand^51^. The mayfly larvae inhabits riverbanks preferring substrates with fine sand or gravel for burrowing and sheltering^51^.

The organisms are collected from two sites on the River Licenza (Latium, Italy) placed within the protected area Lucretili Mountains Park. Samplings occurred from April to July 2019. In total, 44 caddisfly and 121 mayfly larvae were sampled by Surber net sampling in the riverbed and by hand to collect individuals attached under the rocks. All individuals were identified in field and transported in a cool box to the laboratory where caddisflies and mayflies were placed in two different 60 l tanks for one week of acclimation. Then, observations on the natural cases and indoor experiments were conducted (Table 1). Water and sediment exploited in the experiments were collected from the sampling sites.

**Table 1:** Number of individuals (no. ind.) for each species: collected in field, used for observations of natural cases and indoor experiments, and pseudo-replicas experiments.

Species	Number of individuals			
	collected	no. ind. for natural cases	no. ind. for indoor experiments	pseudo-replicas
*Odontocerum albicorne*	44	27	44	8
*Ephemera danica*	121	–	121	34

### Data collection on natural cases

The investigated caddisflies larvae were removed from their natural cases and individuals (Table 1) were photographed, surveyed (in length and width) using the Image Tool 2.0 software, and weighed by a balance (precision 0.1 g). The volume of cases, approximated to a cylinder shape, was calculated from length and width. The density of cases was calculated dividing weight by volume on a subsample of 8 individuals. These data allowed to perform subsequent comparisons with cases made in indoor experiments. In addition, a subsample of 27 natural cases were digested to observe the eventual occurrence of MP. After boiling the hydrogen peroxide (H_2_O_2_, 30%), the solution was left at room temperature for 10 min and then cases were added. The high temperature (60 °C) facilitates the digestion of organic matter occurring in the cases^58^. Then, cases were left in the solution to allow the digestion process to continue at room temperature for 48 h.

After digestion, the case residues were observed by stereomicroscope to identify MP. Specifically, MP abundance, shapes (fragment, fibre and film) and colours were noted. In order to identify the polymer materials, 119 items (26% of the total), randomly chosen, were analysed by Fourier Transform Infrared spectroscopy (FT-IR). In particular, the IR spectra were collected using Thermo scientific Nicolette 6700 spectrophotometer. The spectrum range was 4000–400 cm^−1^ and the resolution of 2 cm^−1^. Chemical composition of polymer particles was identified by comparison with reference spectra database (instrument library and http://www.ftir-polymers.com/soon.htm).

### Indoor experiments

We used different types of lMP polymers for the indoor experiments: fragments of polyethylene terephthalate (PET), polypropylene (PP), polystyrene (PS), acrylonitrile–butadiene–styrene (ABS) and pellets of polyvinylidene fluoride (PVDF).

**Figure 1:**
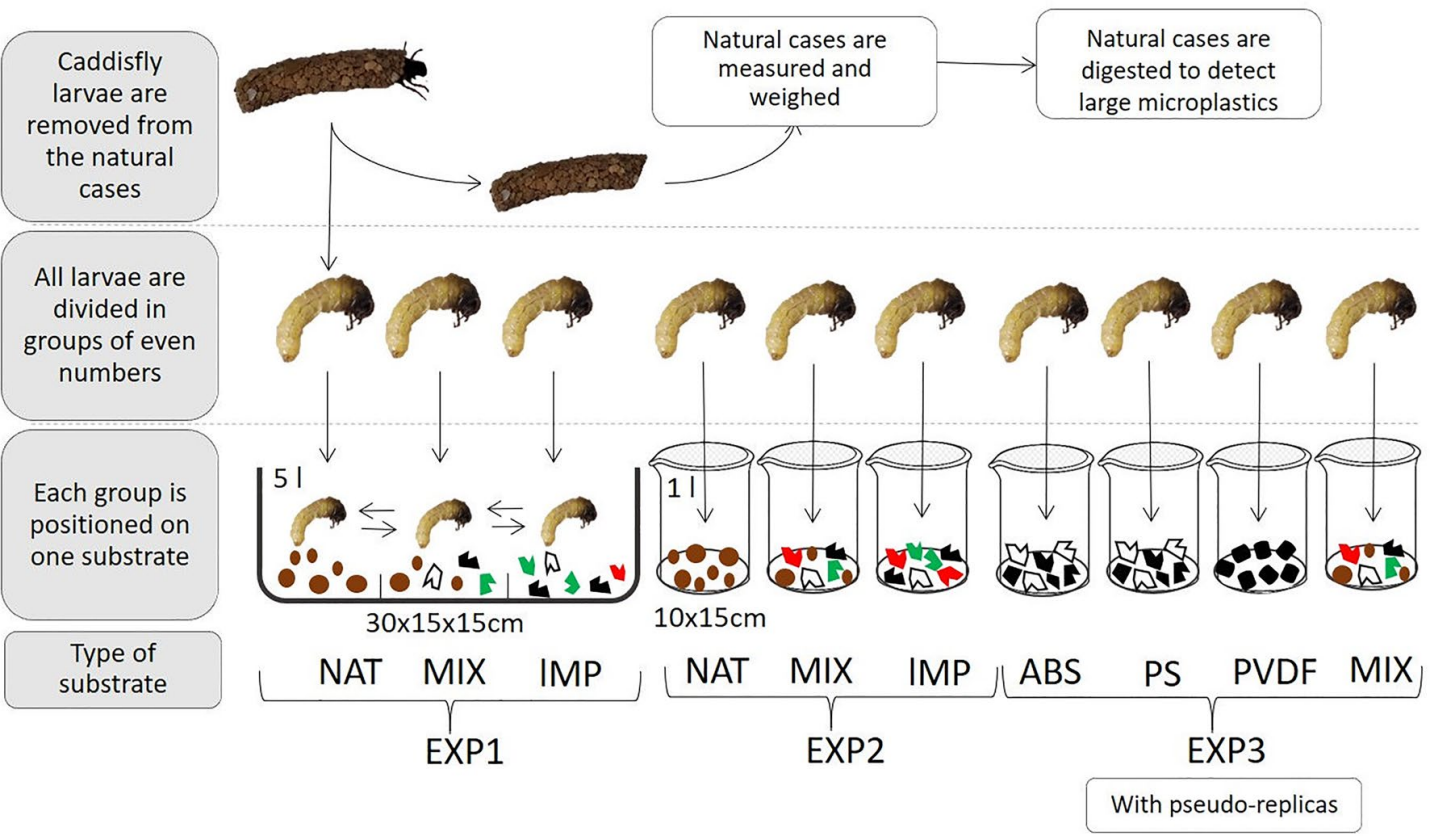
Scheme of the experiments 1 (EXP1), 2 (EXP2) and 3 (EXP3) conducted on caddisflies. The burrowing experiment of mayflies was carried out as EXP1 with the addition of pseudo-replicas. NAT: natural substrate of sand and river pebbles; lMP: mixture of PS, PET, PP (in equal proportions): MIX: mixture of NAT and lMP (50% each); ABS: acrylonitrile–butadiene–styrene; PS: polystyrene; PVDF: polyvinylidene fluoride. Measures of tanks: EXP1 = 30 × 15 × 15 cm (length × width × height), volume 5 l; EXP2 and EXP3 = 10 × 15 cm (diameter × height), volume 1 l. The image is obtained by the software PowerPoint (ver. 2002, https://www.microsoft.com/it-it/microsoft-365/powerpoint).

Three experiments were performed on caddisfly larvae (Fig. 1). The first experiment (EXP1) is conducted in a tank with different substrates, while the second (EXP2) and the third (EXP3) were conducted in a tank with only one type of substrate without and with pseudo-replicas, respectively. The pseudo-replicas are multiple observations on the same analysis unit (i.e. individual)^59^. In detail, in EXP1 27 caddisflies could move freely on three different substrates, divided by small septa in: (1) natural substrate (NAT); (2) substrate with a mixture of lMP (PS, PET, PP in equal proportions); (3) substrate with a mixture of NAT and lMP (MIX, 50% each). Hence, caddisflies were free to move on substrates for making their case. Every 24 h the presence of new cases was checked. When new cases were observed the experiment ended. The new cases built (hereafter artificial cases) were collected and observed by stereomicroscope to evaluate the substrate used. Length, width and volume were obtained as for natural cases (see above).

EXP2 was conducted by placing 9 caddisfly larvae in three tanks, each containing a different substrate: (1) natural substrate (NAT); (2) substrate with a mixture of lMP (PS, PET, PP in equal proportions); (3) substrate with a mixture of NAT and lMP (MIX, 50% each). The presence of new cases was checked at 24 h and 48 h. Length, width and volume were obtained as for natural cases (see above).

Regarding EXP3, 8 caddisflies were placed in 4 tanks, each containing one of the following substrates: 1) ABS; 2) PS; 3) PVDF; 4) mixture of NAT and mix of lMP (PS, PET, PP in equal proportions). After 24 h the artificial cases were collected. Thereafter, the same caddisflies were left in tanks for two more experimental pseudo-replicas during which the artificial cases were collected after 24 h. For all experiments, length, width, volume, weight and density were obtained as for natural cases (see above).

As concern experiments on mayflies, overall 121 mayfly larvae were used, 34 of which were employed also in the 6 pseudo-replicas. Experimental design is the same of EXP1 with the addition of pseudo-replicas. Mayflies were placed for 1 h in the tank to allow individuals to burrow. Then, the number of burrowed mayflies in each substrate was counted, the individuals were photographed, and the body length was measured using the Image Tool 2.0 software. The length was obtained by measuring from head to tail excluding the cerci as in some individuals they were broken^60^.

### Statistical analysis

Normality of data was checked by Shapiro–Wilk test before all analysis. If distribution was not normal, transformation of data was conducted (*e.g.* logarithm) and eventually non-parametric tests were used.

The natural cases of sampled caddisflies were analysed to evaluate the predominance of a specific MP shape and colour by a Kruskal–Wallis test (K-W). Dunn’s post hoc analysis was used to investigate the pairwise difference after each K-W test conducted. The co-occurrence of MP shapes was tested to determine whether pattern was randomic by a Monte-Carlo permutation test. The relationship between volume of natural cases and number of occurring MP was explored by Pearson’s correlation. The measures obtained by natural cases (*i.e.* weight, volume, density) were compared to the ones of artificial cases by unpaired t-tests.

Concerning indoor experiments on caddisflies and mayflies, several tests are used according to specific aims. To investigate whether pseudo-replicas experiments significantly affected the number of artificial cases and mayflies burrowed in substrates, Friedman test was performed. In addition, to evaluate a difference in number of cases or individuals among the three substrates, results were analysed by a K-W test. Dunn’s post hoc analysis was used to investigate the pairwise difference after each K-W test conducted. A heat map was used in order to highlight the results.

Furthermore, the number of mayflies burrowing in natural and MP substrates was investigated to evaluate an eventual significant difference by performing a χ^2^. In addition, to investigate whether the χ^2^ result was affected by the mayfly body length (instead of the type of substrate) a one-way ANOVA was performed. This result was shown with a heat map. All statistical analyses were performed with GraphPad Prism 8.4.2^61^.

## Results

### Microplastics analyses in caddisfly natural cases

**Figure 2:**
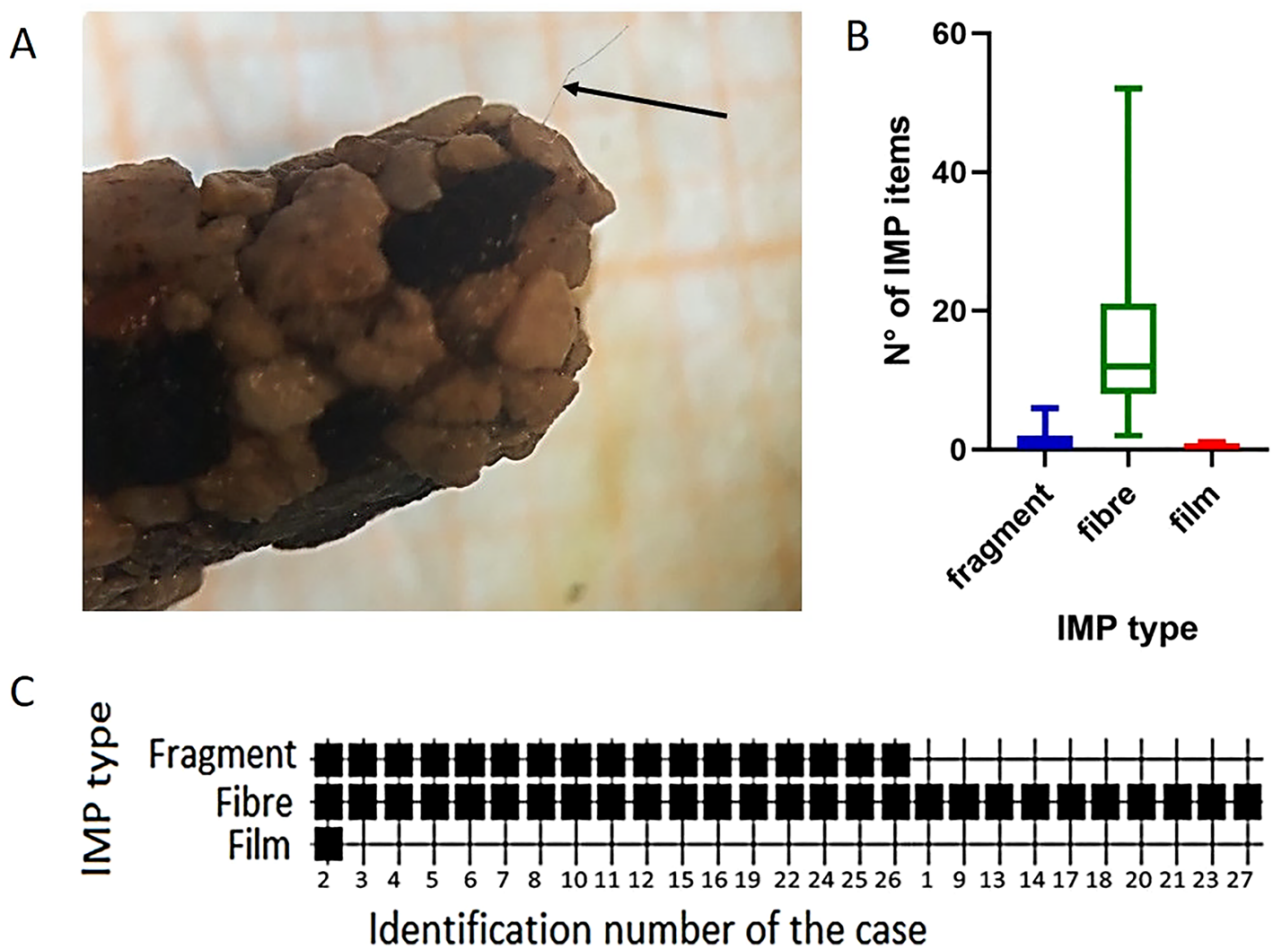
Description of the large microplastics (lMP) found in the caddisfly natural cases. (**A**) The arrow highlights a lMP fibre included in caddisfly natural case. (**B**) Abundance of lMP found in the natural cases of caddisflies. Bars indicate 1–99 percentile. The three groups are significantly different (*p* < 0.01, Kruskal Wallis). (**C**) Black squares on the grid indicate the presence of lMP divided per shape (fragment, fibre, film) in each natural case of caddisflies. The image (**A**) is obtained by the software PowerPoint (ver. 2002, https://www.microsoft.com/it-it/microsoft-365/powerpoint); (**B**) by the software GraphPad Prism (ver. 8.4.2, https://www.graphpad.com/support/faq/prism-842-release-notes/), (**C**) by the software PAST (ver. 3.14, http://folk.uio.no/ohammer/ past/)^61^.

MP were found in the cases of caddisfly collected from River Licenza (Fig. 2A). MP belonged all to the category of lMP. A total of 458 lMP were found in 27 natural cases of caddisfly with a mean of 17 lMP/case (Supplementary Information Table S1, S2). The number of lMP items was slightly positively correlated to the volume of the cases (r = 0.42, *p* < 0.05).

According to shape, lMP belonged to three different categories: fragments, fibres and films. Fragments were present in 17 cases (63%), all 27 cases contained fibres (100%), and 1 case contained film (4%). Fibres were the most abundant lMP shape in natural cases (H = 64.67, df = 52, *p* < 0.01) (Fig. 2B). Dunn’s post hoc test confirmed the significant differences between the three groups (*p* < 0.05).

Moreover, only 1 case (4%) contained all the three plastic shapes, while 16 cases (59%) contained both fibres and fragments and 10 cases (37%) only fibres (Fig. 2C). The analysis of the co-occurrence of lMP shapes in cases pointed out the presence of a pattern, because the data of presence was not randomly distributed (*p* < 0.01).

**Figure 3:**
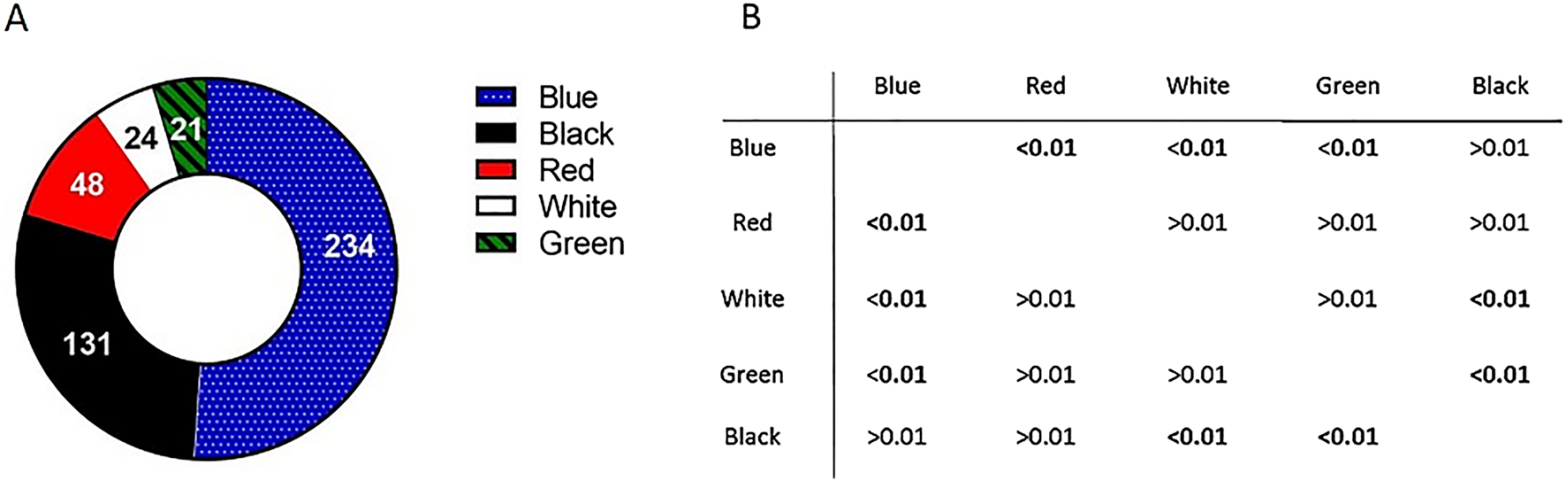
(**A**) Abundances of large microplastics (lMP) items by colour observed in the cases of caddisflies sampled from River Licenza (Latium, Italy). (**B**) In the table, *p* values of Dunn’s post hoc test show the pairwise comparison of the abundances of lMP items of different colours. Significant values (Kruskal Wallis, *p* < 0.01) are in bold. The images (**A**) and (**B**) are obtained by the software GraphPad Prism (ver. 8.4.2, https://www.graphpad.com/support/faq/prism-842-release-notes/).

The detected colours of the lMP items were blue, red, white, green and black (Fig. 3A). Blue was the predominant colour of both fibres and fragments. The only film item found was white. The difference between colour abundances was significant (H = 42.77, df = 216, *p* < 0.01). Although Dunn’s post hoc test revealed that the abundances of blue and black items did not significantly differ from each other, they were significantly more abundant than the other colours (Fig. 3B).

The analysis of the polymeric materials by FT-IR spectroscopy showed the presence of synthetic fibres such as polyester (40% of the total) and polyamides (PA; 32% of the total) (Supplementary Information Fig. S1). Regarding fragments, the polyethylene (PE) and polypropylene (PP) are the most abundant polymers identified in caddisfly cases, 30% and 19% respectively (Supplementary Information Fig. S2).

### Indoor experiments on caddisflies

**Figure 4:**
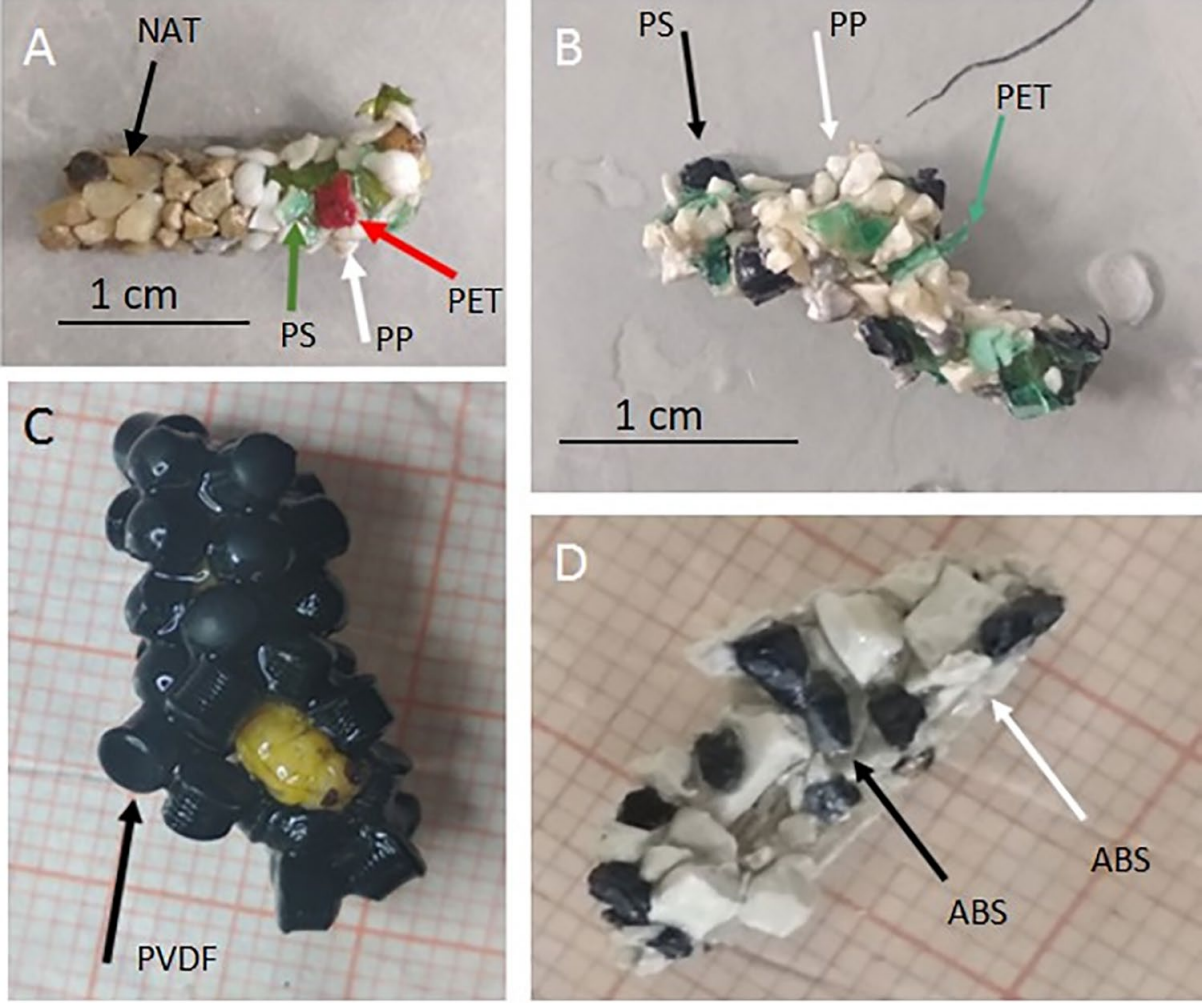
Artificial cases built by caddisfly larvae in indoor experiments using different substrates: (**A**) MIX = natural substrate (NAT) and lMP (50% each); (**B**) lMP = PET, PP, PS (in equal proportions); (**C**) PVDF = polyvinylidene fluoride; and (**D**) ABS = acrylonitrile butadiene styrene. The arrows indicate different substrates. The images (**A**), (**B**), (**C**) and (**D**) are obtained by the software PowerPoint (ver. 2002, https://www.microsoft.com/it-it/microsoft-365/powerpoint).

Based on indoor experiments, caddisflies can build new cases with all the provided lMP polymers and shapes (Fig. 4; Supplementary Information Table S3, S4, S5).

**Figure 5:**
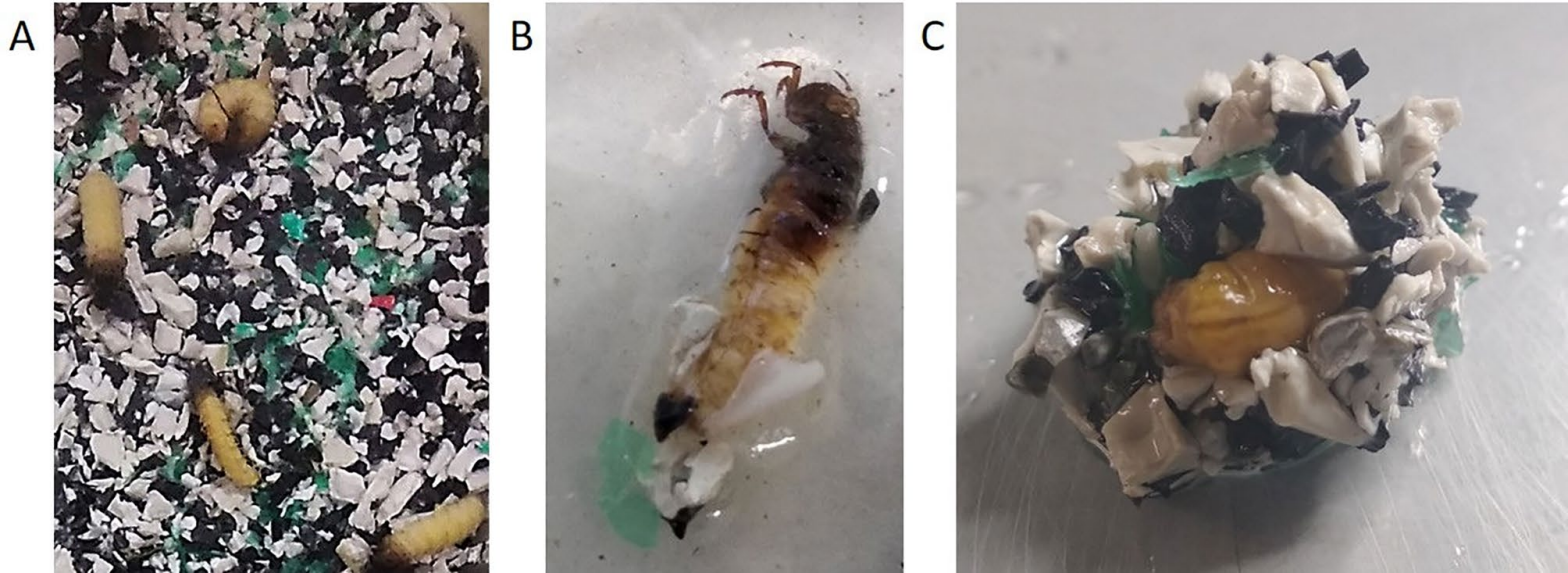
The case rebuilding during the experiment 1 (EXP1). (**A**) Caddisflies exposed to substrate with a mixture of lMP (PS, PET, PP in equal proportions); (**B**) initial phase of case rebuilding with lMP; (**C**) case rebuilt observed from dorsal vision. The images (**A**), (**B**) and (**C**) are obtained by the software PowerPoint (ver. 2002, https://www.microsoft.com/it-it/microsoft-365/powerpoint).

As EXP1 and EXP2 concern, caddisfly larvae rebuilt their cases using MIX and lMP substrates. In particular, in EXP1 15% of larvae rebuilt their cases and used MIX and lMP substrates (Fig. 5). In EXP2 33% of larvae rebuilt their cases and used only lMP substrate.

**Figure 6:**
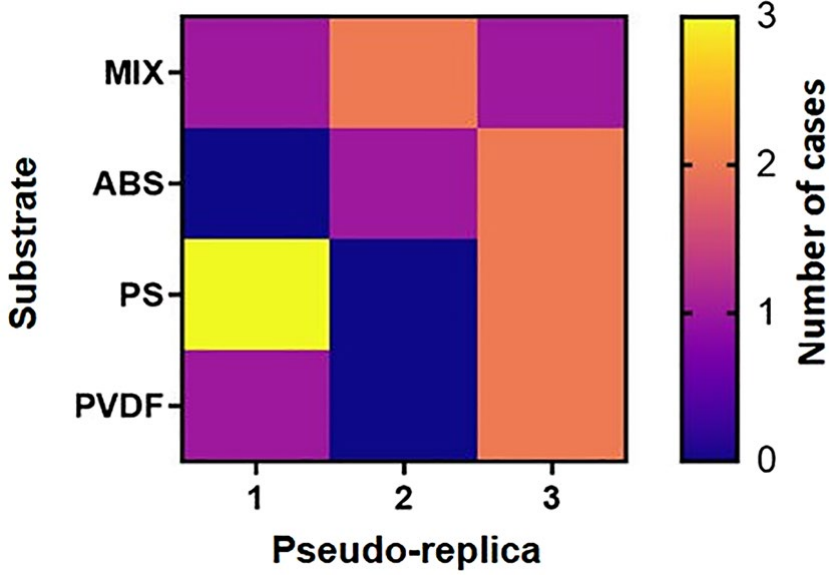
Heat map with the number of artificial cases rebuilt by caddisflies in each substrate for the three pseudo-replicas. Replica no. 1 indicate the first time of exposure. MIX = natural substrate and lMP (50% each); ABS = acrylonitrile butadiene styrene; PS = polystyrene; PVDF = polyvinylidene fluoride. The image is obtained by the software GraphPad Prism (ver. 8.4.2, https://www.graphpad.com/support/faq/prism-842-release-notes/).

Regarding EXP3, PS was the most utilized polymer to build artificial cases, followed in decreasing order by mixed substrate, PVDF and ABS. There was no significant difference between the number of cases in the four lMP substrates. Regarding the results of the three pseudo-replicas, there was no significant change in number of artificial cases built (Fig. 6).

### Comparison between natural and artificial cases

**Figure 7:**
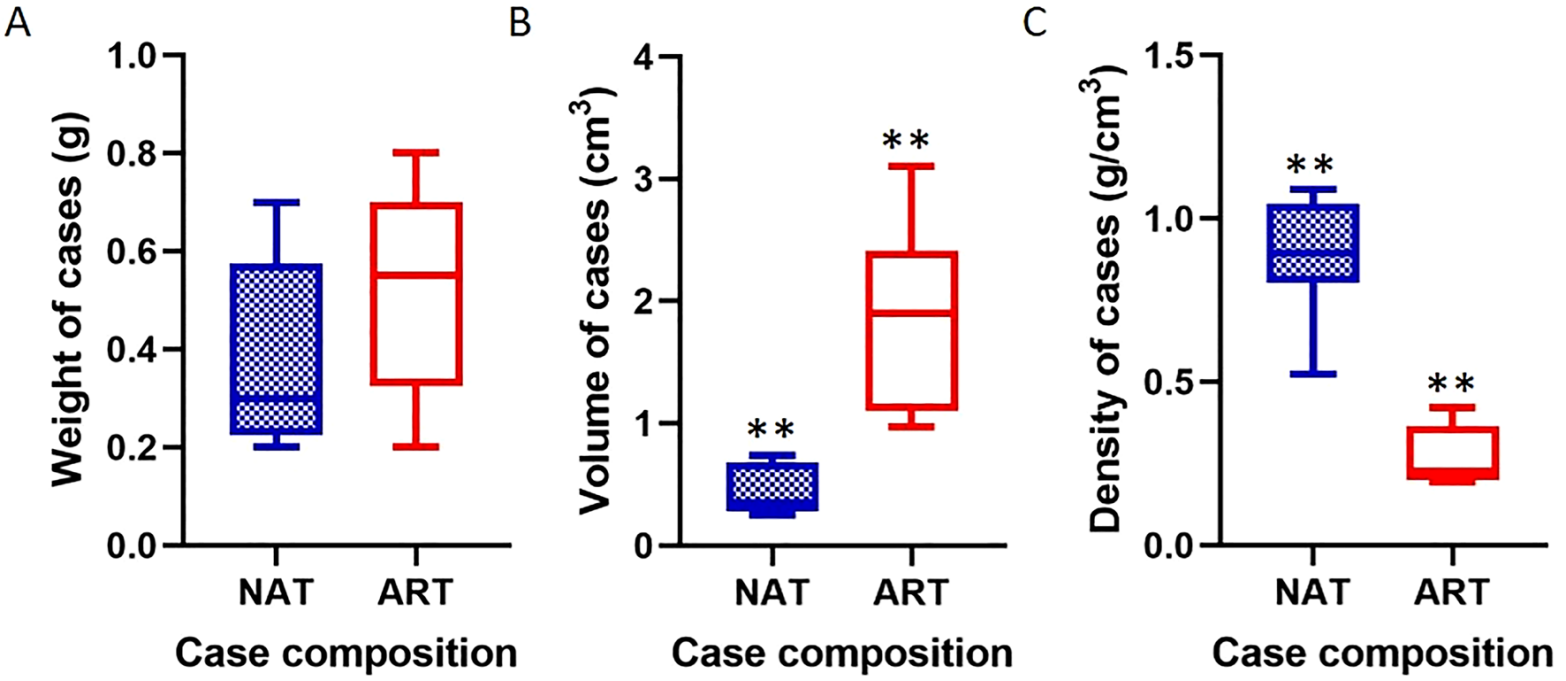
(**A**) Weight, (**B**) volume and (**C**) density comparisons between natural and artificial cases built by caddisflies. Bars indicate 1–99 percentile in the box plot. NAT = cases built with natural substrate; ART = artificial cases built with MIX and lMP substrates. The volume and the density between natural and artificial cases are significantly different (**p < 0.01, unpaired t-test). The images (**A**), (**B**) and (**C**) are obtained by the software GraphPad Prism (ver. 8.4.2, https://www.graphpad.com/support/faq/prism-842-release-notes/).

The weight of artificial cases did not differ significantly from the natural ones (Fig. 7A). Instead, the volume of artificial cases was significantly higher by about 4 times (t = 5.353, df = 13, *p* < 0.01) (Fig. 7B). Concerning the density, there was a significant difference between natural and artificial cases (t = 8.044, df = 13, *p* < 0.01). The mean density of artificial cases was 0.27 times the natural one (Fig. 7C).

### Indoor experiments on mayflies

**Figure 8:**
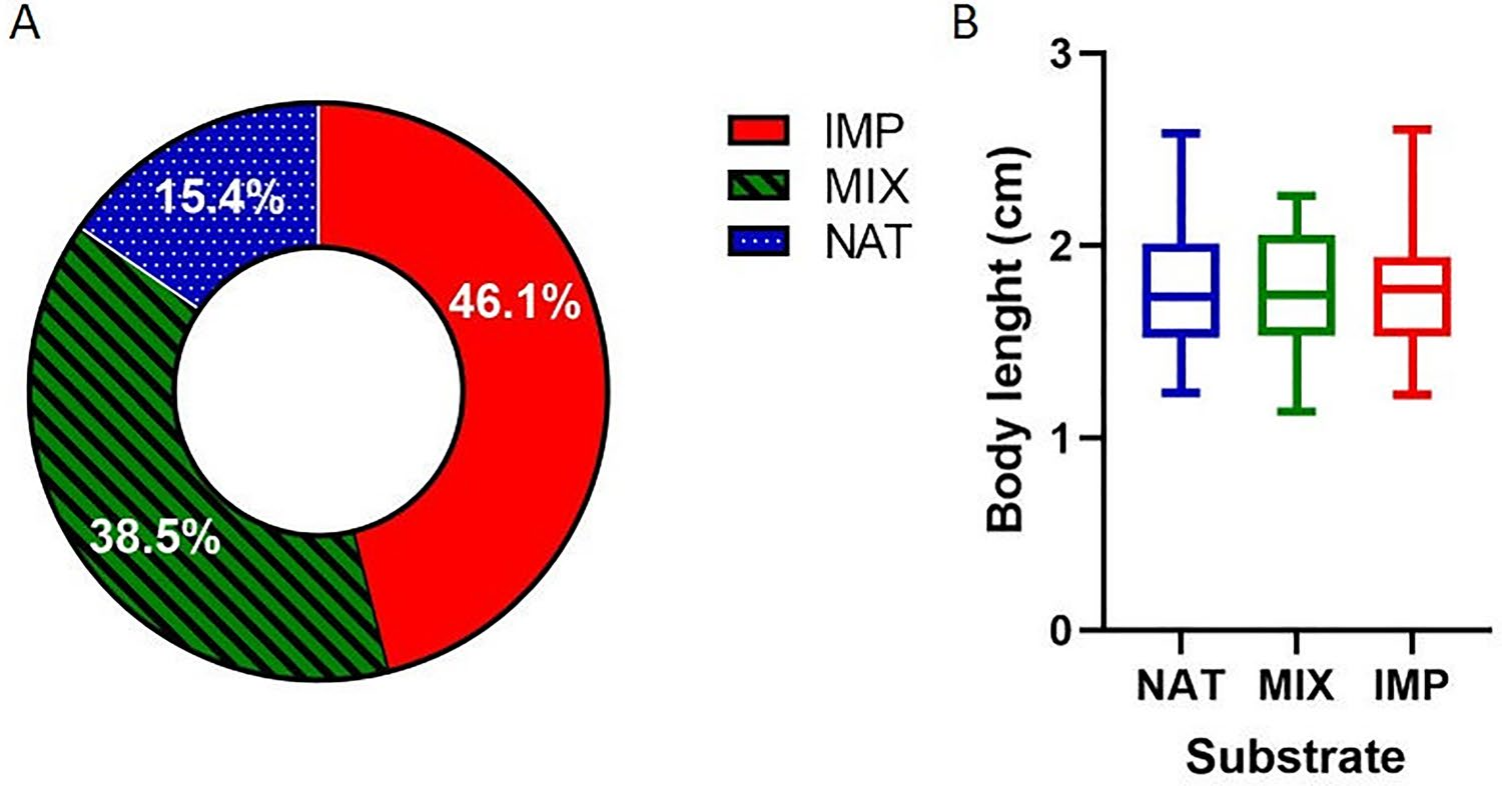
(**A**) Colonization of substrates by mayflies. (**B**) Body length for each substrate. Bars indicate 1–99 percentile in the box plot. NAT = natural substrate; lMP = PET, PP, PS (in equal proportions); MIX = NAT and lMP (50% each). The images (**A**) and (**B**) are obtained by the software GraphPad Prism (ver. 8.4.2, https://www.graphpad.com/support/faq/prism-842-release-notes/).

Individuals of mayflies colonized more lMP substrate (46.1%) than mixture of natural and lMP substrate (38.5%) and natural substrate (15.4%) (χ^2^ = 29.50, df = 16, *p* < 0.05) (Fig. 8A). The body length difference was not significant between the three substrates (Fig. 8B; Supplementary Information Table S6).

**Figure 9:**
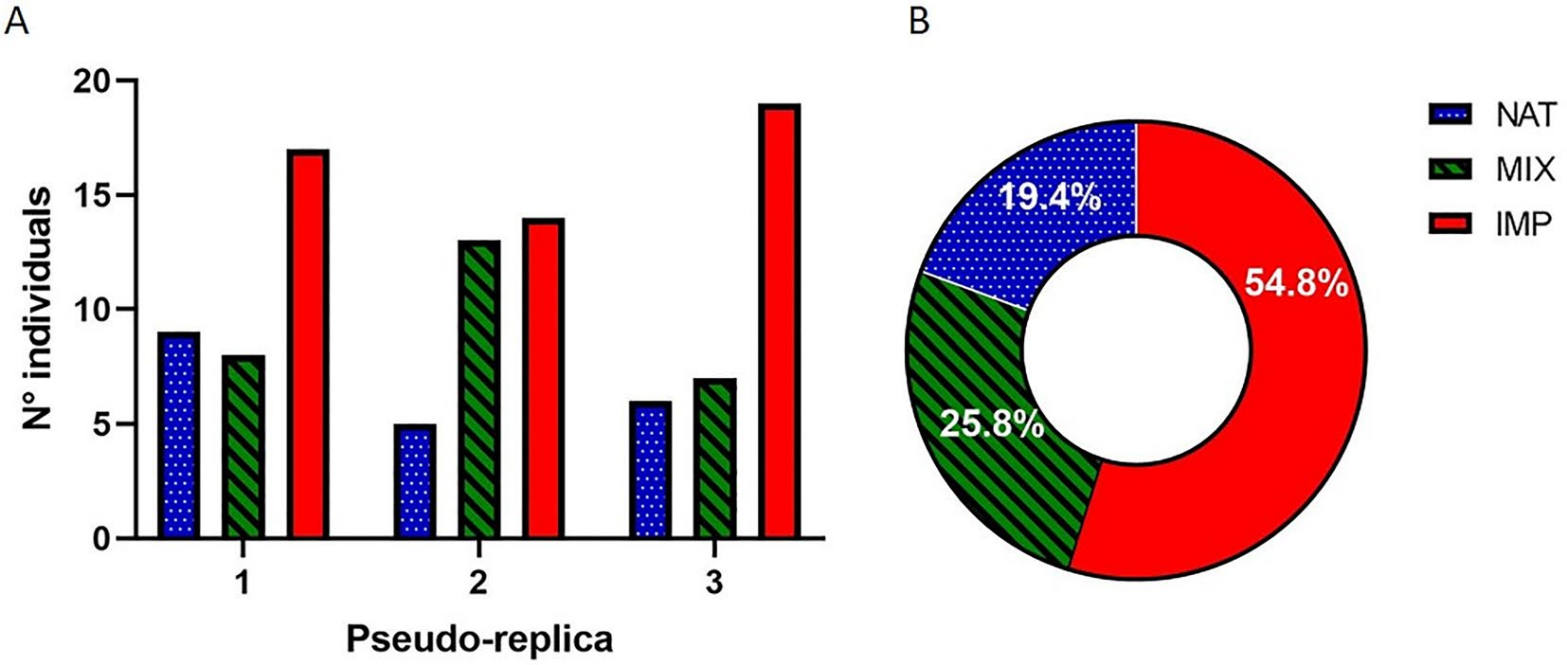
Results on occurrence of mayflies in each substrate for pseudo-replicas experiments: (**A**) results are shown for each pseudo-replica; (**B**) results of all pseudo-replicas are summed. Replica no. 1 indicate the first time of exposure. NAT = natural substrate; lMP = PET, PP, PS (in equal proportions); MIX = NAT and lMP (50% each). The images (**A**) and (**B**) are obtained by the software GraphPad Prism (ver. 8.4.2, https://www.graphpad.com/support/faq/prism-842-release-notes/).

Concerning the experimental pseudo-replicas, the occurrence of individuals in substrates did not show significant changes in relation to previous exposure to the test (Fig. 9A). In all pseudo-replicas, the lMP substrate was more colonized by mayflies (H = 5.956, df = 4, *p* < 0.05) (Fig. 9B). Dunn’s post hoc analysis confirmed the significant difference only between the number of mayflies burrowing in natural and lMP substrates (z = 2.385, *p* < 0.05).

## Discussion

Research on macroinvertebrates is ongoing and rapidly expanding, although the effects of MP on freshwater benthic community have been poorly investigated rather than other taxonomic groups such as fish (see^11^ and references therein). Ingestion of MP is one of the main topics of investigation; however, here we focused on understudied and innovative aspects on the impacts of MP on macroinvertebrates.

The effects of MP are investigated in two macroinvertebrates species not previously studied: the caddisfly *Odontocerum albicorne* and the mayfly *Ephemera danica*. We report the first evidence of MP presence in cases of caddisfly and the capacity of its larvae to build artificial cases with different commercial plastic polymers. Furthermore, we compare for the first time the weight, volume, and density of natural and artificial cases. In addition, the burrowing behaviour of mayflies in plastic substrates is investigated for the first time. In particular, we find that mayflies burrows mainly in artificial substrates.

### Case making caddisflies in nature and indoor conditions

We contributed to research MP effects on caddisflies by observing the inclusion of MP in the cases of *O. albicorne*. To date, only two other works report MP inclusion in caddisfly cases in freshwaters^53,54^ (see “Introduction”).

The characteristics of the MP incorporated in natural cases of *O. albicorne* were compared to the ones of *Lepidostoma basale* (Lepidostomatidae^54^). Regarding MP colours, blue is the most abundant in both species*.* It is unsure if this shows a preference of the organisms for this colour or rather its environmental bioavailability. It is known that polarised light may influence the behaviour of adult macroinvertebrates^62^, however, further researches are needed to study the response of larvae to colours.

Regarding the comparison of MP shapes, *O. albicorne* cases have more fibres than films and fragments, while the opposite is observed for *L. basale* cases. Regarding the polymers, polyester and PA are the most common fibres found into the natural cases of *O. albicorne* as also observed in *L. basale*^54^. The presence of polyester and PA fibres is probably due to the discharge of a wastewater treatment plant upstream of the sampling point^63,64^. In both caddisfly species PP fragments are found, which is not surprising as PP represents one of the most abundant polymers detected in biota^11^. MP found in cases are suggested to depend on the environmental bioavailability where caddisflies inhabit^54^. Other factors such as case volume limitedly explain the abundance of MP, based on the results of our correlation test. As MP in cases could be a proxy of their occurrence in freshwaters, further investigations could assess whether the use of the cases could contribute to environmental detection of MP contamination.

*O. albicorne* larvae never built cases using the natural substrate only, even if they could use both natural and artificial substrate. In fact, it seems that larvae used more lMP substrate. In addition, experimental data reveal that *O. albicorne* can use the bioavailable MP as construction material independently of polymer, shape and pseudo-replica. The capacity of this species to include different MP could enhance its resilience. In fact, they may camouflage their cases even in disturbed freshwaters due to plastic contamination. However, toxic effects could affect the organisms due to prolonged plastic exposure. Moreover, negative effects are reported on the stability of the structure of the cases that include plastics^54^. There is still a wide knowledge gap on the impacts of MP on caddisflies.

### Burrowing mayflies occurrence in natural and microplastic substrates

In this work, for the first time the mayfly *E. danica* was used to evaluate the burrowing in natural and microplastic substrates. The nature of the substrate is a main physical factor determining the distribution of mayflies in the running waters. In fact, the burrowing mayflies dig U-shaped tunnels in the bottom with fine grain size (sand, silt) or wedge in the sediments that accumulate in the cracks between the stones^51^. In this study, mayflies colonize more artificial substrates over the natural substrate in both one exposure and pseudo-replicas experiments. This observation could be explained by the fact that the plastic used is lighter than the natural substrate and therefore the mayflies can burrow more easily and hide from eventual predators more quickly.

As the occurrence in each substrate is not related to body length of mayflies, the presence of more individuals burrowed in artificial substrate compared to natural one is probably occurring independently of the larval stage of animals. This suggests that mayflies may be highly exposed to lMP in contaminated natural environments. Indeed, the ingestion of MP by Ephemeridae (Baetidae, Heptageniidae) was proven in riverine habitats^44^. Although effects due to ingestion are not studied in mayflies, negative impacts were observed in other macroinvertebrates (*e.g.* larval growth and imagoes emergence^65^).

## Conclusion

The recent impacts of microplastics in the Anthropocene era is likely to represent a challenge for the riverine habitats^66^. The present work has contributed to expand the research on the effects of plastics on aquatic biota by investigating the case making of caddisflies and the burrowing behaviour of mayflies.

In this preliminary study, we showed the capacity of the caddisfly *Odontocerum albicorne* and the mayfly *Ephemera danica* to use microplastics when included in the substrates. Natural and indoor observations suggest that these species are resilient and can live in an environment contaminated by microplastics, using them as construction material for cases and as substrate where to burrow. Moreover, caddisflies and mayflies seem to use mainly microplastics over the natural construction material or substrate, thus highlighting a possible attraction for the microplastic substrates. Further researches are mandatory to investigate the causes of larvae attraction to plastics. In this regard, the colour of plastics is suggested to be considered when addressing the larvae attraction to plastics, since our results showed that caddisflies used mainly blue microplastics for making their cases.

In addition, the impacts of plastics on caddisflies and mayflies are suggested to be further investigated by scientific literature in accordance to the high use of microplastics by larvae that is highlighted in this work. For example, we highlight a knowledge gap on the plastic impacts on drift behaviour of macroinvertebrates. In fact, a higher plastic occurrence in the cases of caddisflies and in substrates where the mayflies burrow could increase drift of larvae.

In conclusion, researches on caddisflies and mayflies are encouraged to increase knowledge on the impacts of plastic pollution on macroinvertebrates and to evaluate the potential of caddisflies cases as suitable microplastics monitoring tools to assess the riverine contamination by microplastics.

## Supplementary Information

Supplementary Information.

## Data Availability

All data generated or analysed during this study are included in this published article (and its Supplementary Information files).
