# Supplementary material for: Preliminary indoor evidences of microplastic effects on freshwater benthic macroinvertebrates
Source: Sci Rep. 2021 Jan 12;11:720. doi: 10.1038/s41598-020-80606-5 (PMC7803787; doi:10.1038/s41598-020-80606-5)
Supplement: Supplementary file 1 — Supplementary Information. [file 41598_2020_80606_MOESM1_ESM.pdf]

Supplementary Information

Preliminary indoor evidences of microplastic effects on freshwater benthic macroinvertebrates

Luca Gallitelli[1], Alessandra Cera[1], Giulia Cesarini[1]*, Loris Pietrelli[2], Massimiliano Scalici[1]

Authors' addresses:

[1] University of Roma Tre, Department of Sciences, Viale G. Marconi 446, 00146 Rome, Italy

[2] Sapienza University of Rome, Department of Chemistry, P.le A. Moro, 5, 00185 Rome, Italy

* Corresponding author: giulia.cesarini@uniroma3.it

Content

Table S1. Biometries (length, width, weight, volume) of natural cases and number of large microplastics (lMP) occurred in *Odontocerum albicorne* cases.

Table S2. Colour and shape of large microplastics (fragments, fibres, films) occurred in each natural case of *Odontocerum albicorne*.

Figure S1. FTIR spectrum of polyester fiber found in natural cases of *Odontocerum albicorne*.

Figure S2. Relative amount (%) of microplastic polymer materials (polyamide; polyester; polyacrylic; PE: polyethylene; PP: polypropylene; PS: polystyrene; PVC: polyvinylchloride) of fragments and fibres found in natural case of *Odontocerum albicorne*.

Table S3. Biometries (length, width, volume) of artificial cases rebuilt by *Odontocerum albicorne* during experiment 1 (EXP1).

Table S4. Biometries (length, width, volume) of artificial cases rebuilt by *Odontocerum albicorne* during experiment 2 (EXP2).

Table S5. Biometries (length, width, volume, weight, density) of artificial cases rebuilt by *Odontocerum albicorne* during experiment 3 (EXP3). n.a.= measures not available.

Table S6. Body length (cm) of *Ephemera danica* burrowed in different substrates. NAT = natural substrate, MIX = mixture of natural and microplastic substrates, lMP = microplastic substrate.

Table S1. Biometries (length, width, weight, volume) of natural cases and number of large microplastics (lMP) occurred in *Odontocerum albicorne* cases.

Natural cases					
**caddisfly ID**	**lenght (cm)**	**width (cm)**	**weight (g)**	**volume (cm^3)**	**number of lMP**
1	1.83	0.63	0.2	0.57	26
2	2.55	0.78	0.4	1.22	54
3	2.62	0.66	0.3	0.90	51
4	2.41	0.58	0.3	0.64	28
5	2.39	0.58	0.2	0.63	13
6	2.16	0.60	0.3	0.61	41
7	1.99	0.81	0.5	1.02	13
8	1.94	0.64	0.2	0.62	16
9	2.65	0.67	0.3	0.93	9
10	2.27	0.59	0.2	0.62	10
11	1.99	0.69	0.2	0.74	29
12	2.75	0.61	0.2	0.80	12
13	1.92	0.52	0.1	0.41	2
14	2.13	0.62	0.2	0.64	5
15	2.13	0.66	0.3	0.73	11
16	1.94	0.54	0.2	0.44	5
17	2.28	0.68	0.4	0.83	13
18	2.23	0.61	0.3	0.65	3
19	2.43	0.66	0.3	0.83	12
20	2.65	0.61	0.4	0.77	9
21	2.44	0.57	0.4	0.62	4
22	2.84	0.74	0.4	1.22	10
23	2.68	0.66	0.4	0.92	21
24	2.48	0.64	0.3	0.80	21
25	2.55	0.59	0.4	0.70	10
26	1.86	0.52	0.1	0.39	14
27	2.83	0.58	0.4	0.75	16

Table S2. Colour and shape of large microplastics (fragments, fibres, films) occurred in each natural case of *Odontocerum albicorne*.

Natural cases											
**caddisfly ID**	**fragments**					**fibres**					**films**
	**blue**	**red**	**white**	**green**	**black**	**blue**	**red**	**white**	**green**	**black**	**white**
1	0	0	0	0	0	10	4	0	1	11	0
2	1	0	0	0	0	40	7	0	0	5	1
3	2	0	0	0	0	27	7	0	1	14	0
4	1	0	5	0	0	11	2	0	4	5	0
5	0	0	1	0	0	4	0	0	3	5	0
6	0	0	0	1	0	14	6	1	7	12	0
7	0	0	0	1	0	9	1	0	0	2	0
8	2	0	0	0	0	6	1	0	0	7	0
9	0	0	0	0	0	5	0	3	1	0	0
10	0	0	0	0	1	5	1	0	0	3	0
11	0	0	1	0	0	14	3	4	1	6	0
12	0	0	1	0	0	7	0	0	0	4	0
13	0	0	0	0	0	2	0	0	0	0	0
14	0	0	0	0	0	0	0	0	0	5	0
15	0	0	2	0	0	2	1	0	0	6	0
16	0	0	2	0	0	0	0	0	1	2	0
17	0	0	0	0	0	3	2	0	0	8	0
18	0	0	0	0	0	2	0	0	0	1	0
19	1	0	0	0	1	8	0	0	0	2	0
20	0	0	0	0	0	3	1	1	0	4	0
21	0	0	0	0	0	1	0	0	0	3	0
22	2	0	0	0	0	5	0	0	0	3	0
23	0	0	0	0	0	17	2	0	0	2	0
24	1	1	0	0	0	13	3	0	0	3	0
25	2	0	0	0	0	2	3	1	0	2	0
26	1	0	0	0	0	6	2	0	0	5	0
27	0	0	0	0	0	5	1	1	0	9	0
**total**	13	1	12	2	2	221	47	11	19	129	1

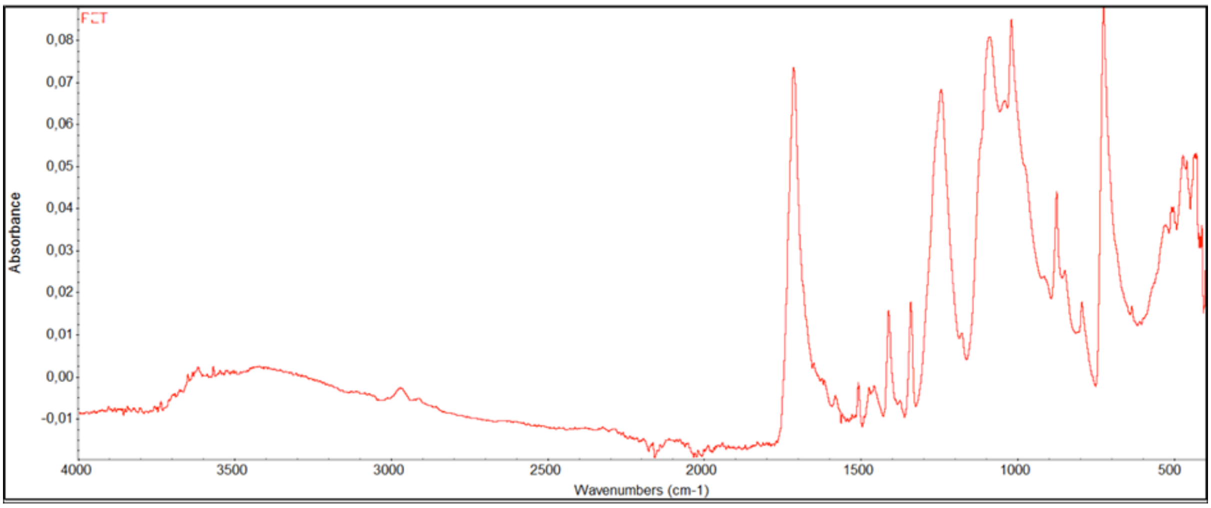

Figure S1. FTIR spectrum of polyester fiber found in natural cases of *Odontocerum albicorne*.

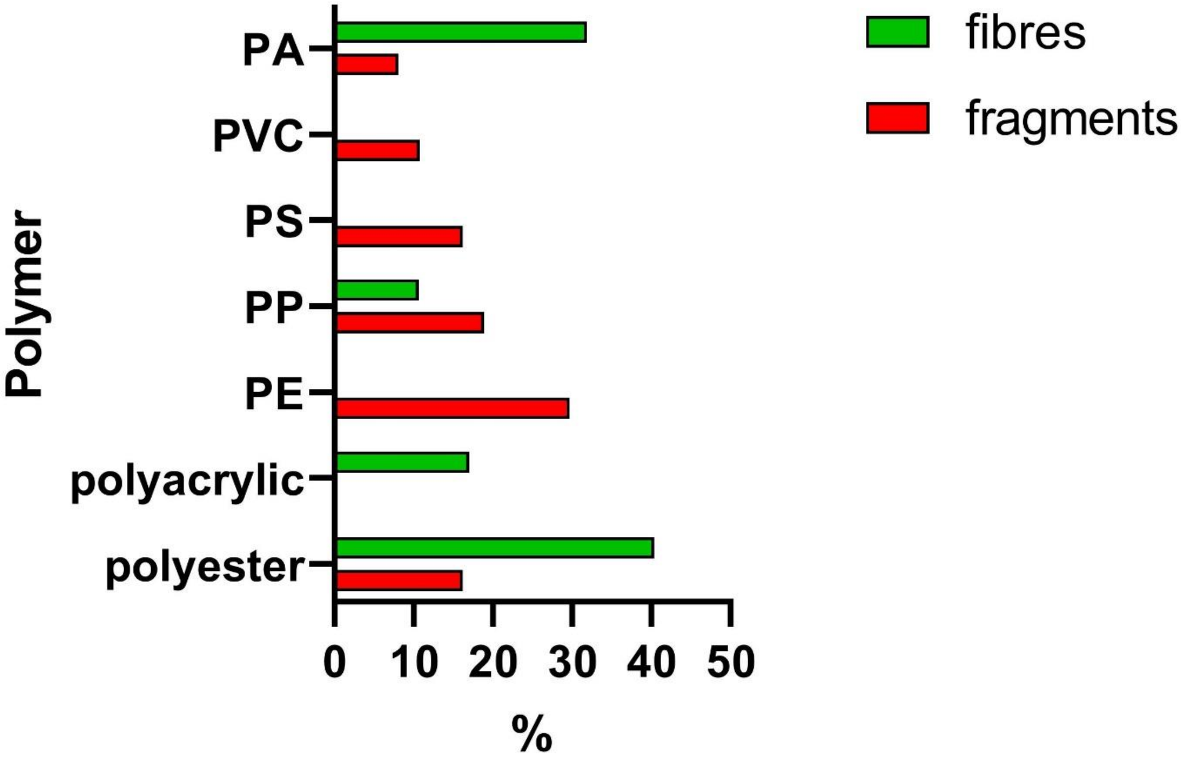

Figure S2. Relative amount (%) of microplastic polymer materials (polyester; polyacrylic; PE: polyethylene; PP: polypropylene; PS: polystyrene; PVC: polyvinylchloride; PA: polyamide) of fragments and fibres found in natural cases of *Odontocerum albicorne*.

Table S3. Biometries (length, width, volume) of artificial cases rebuilt by *Odontocerum albicorne* during experiment 1 (EXP1).

Artificial cases EXP1			
**caddisfly ID**	**lenght (cm)**	**width (cm)**	**volume (cm^3)**
1	0.50	0.28	0.03
2	0.72	0.44	0.11
3	1.33	0.34	0.12
4	0.50	0.40	0.06

Table S4. Biometries (length, width, volume) of artificial cases rebuilt by *Odontocerum albicorne* during experiment 2 (EXP2).

Artificial cases EXP2			
**caddisfly ID**	**lenght (cm)**	**width (cm)**	**volume (cm^3)**
1	0.81	0.48	0.15
2	0.45	0.36	0.05
3	0.39	0.17	0.01

Table S5. Biometries (length, width, volume, weight, density) of artificial cases rebuilt by *Odontocerum albicorne* during experiment 3 (EXP3). n.a.= measures not available.

Artificial cases EXP3					
**caddisfly ID**	**lenght (cm)**	**width (cm)**	**volume (cm^3)**	**weight (g)**	**density (g/cm^3)**
1	1.35	1.29	1.76	0.4	0.23
2	2.03	1.23	2.41	0.5	0.21
3	1.37	0.95	0.97	0.3	0.31
4	3.15	1.12	3.10	0.6	0.19
5	2.19	1.05	1.90	0.8	0.42
6	2.37	1.03	1.97	0.7	0.35
7	n.a.	n.a.	n.a.	0.7	n.a.
8	1.85	0.87	1.10	0.2	0.18

Table S6. Body length (cm) of *Ephemera danica* burrowed in different substrates. NAT = natural substrate, MIX = mixture of natural and microplastic substrates, lMP = microplastic substrate.

Body length of mayflies occurred in each substrate		
**NAT**	**MIX**	**lMP**
1.23	1.14	1.22
1.41	1.29	1.24
1.44	1.35	1.30
1.45	1.40	1.33
1.51	1.42	1.33
1.52	1.46	1.39
1.55	1.50	1.42
1.62	1.52	1.44
1.62	1.52	1.46
1.63	1.53	1.47
1.66	1.54	1.52
1.73	1.56	1.53
1.73	1.57	1.53
1.75	1.61	1.56
1.80	1.67	1.59
1.81	1.69	1.60
1.84	1.69	1.62
2.01	1.71	1.62
2.01	1.72	1.64
2.02	1.76	1.65
2.12	1.8	1.68
2.40	1.80	1.71
2.58	1.82	1.77
-	1.85	1.77
-	1.93	1.77
-	1.98	1.78
-	1.99	1.81
-	2.01	1.81
-	2.05	1.81
-	2.07	1.91
-	2.15	1.91
-	2.16	1.92
-	2.16	1.93
-	2.19	1.93
-	2.21	1.95
-	2.21	1.97
-	2.22	2.08
-	2.26	2.11
-	-	2.22
-	-	2.27
-	-	2.27
-	-	2.29
-	-	2.34
-	-	2.54
-	-	2.60
